# Exploring of therapeutic potential of indirubin-loaded nanofibrous scaffolds for localized melanoma treatment

**DOI:** 10.1186/s13036-025-00598-6

**Published:** 2025-12-18

**Authors:** Sajad Rahimi, Kamyar Khoshnevisan, Amir Hossein Izadi Nazar, Maryam Doostan, Hassan Maleki

**Affiliations:** 1 Student Research Committee, Kermanshah University of Medical Sciences, Kermanshah, Iran; 2 Pharmaceutical Sciences Research Center, Health Institute, Kermanshah University of Medical Sciences, Kermanshah, Iran; 3 Medical Nanotechnology and Tissue Engineering Research Center, Shahid Beheshti University of Medical Sciences, Tehran, Iran; 4 Research and Development Team, Evolution Wound Dressing (EWD) Startup Co, Tehran, Iran

**Keywords:** Nanofibrous scaffold, Indirubin, Melanoma, Cell viability, Apoptosis

## Abstract

Nanofibrous scaffolds have been established as capable platforms for localized drug delivery systems of skin cancer management. Our study aimed to develop an electrospun nanofibrous scaffold loaded with indirubin, a naturally occurring bisindole alkaloid found in plants that bear indigo, for efficient targeted chemotherapy. The scaffold was prepared from polyvinyl alcohol (PVA) and chitosan (CS), using the electrospinning process, and incorporated with indirubin. Additional experiments were conducted to evaluate the physicochemical properties, perform antioxidant assays, and analyze the release profile. The scaffolds’ anticancer activity was evaluated on human skin melanoma cells. The prepared indirubin-loaded PVA/CS scaffold exhibited nanofibers (NFs) with a nanoscale diameter, a defect-free, and homogeneous morphology. Infrared spectroscopy confirmed the successful incorporation of indirubin into the scaffold. The scaffold displayed a hydrophilic surface, sufficient mechanical strength, high porosity, and a high liquid absorption capacity. In addition, the incorporation of 15 wt% indirubin demonstrated notable antioxidant properties, and its release from the NFs occurred in a controlled and extended manner over approximately 72 h. Furthermore, the scaffold significantly declined cell viability and induced apoptosis in melanoma cells. It is believed that an indirubin-loaded PVA/CS nanofibrous scaffold holds promise as a valuable system for localized melanoma treatment.

## Introduction

Skin cancer, the predominant form of malignancy worldwide in both melanoma and non-melanoma skin cancers (NMSC), has seen a significant increase over the past few decades [1]. In 2024, the American Cancer Society estimated that 100,640 new cases of melanoma were reported [2]. The uncontrolled growth of abnormal skin cells, a hallmark of skin cancer, is primarily attributed to unaddressed DNA damage. This damage leads to genomic abnormalities and rapid cell proliferation, ultimately culminating in the development of cancerous tumors [3, 4]. Due to the challenges mentioned above, scientists have considered various management approaches for skin cancer.

Different treatment interventions have been applied to eliminate skin and tumor lesions. Nowadays, numerous therapies are exploited to relieve the symptoms of skin cancer. These systems of therapy include chemotherapy, radiation therapy, immunotherapy, and surgical removal of the tumor. The choice of therapeutic mode depends on factors such as the tumor’s accessibility, stage of advancement, borders, and size [5]. The primary approach to skin cancer management involves surgical interventions aimed at completely removing the lesion to prevent its growth and spread, while preserving its aesthetic appearance and functional capabilities. However, recurrence and containment are often not possible [6]. The challenge of effectively treating potentially hazardous tumors in skin cancer persists despite the approval of numerous pharmaceuticals by the Food and Drug Administration, as their efficiency is restricted. One potential explanation for this phenomenon is the complex structure of both the skin layers and cancerous tissue that hinders the effective administration and penetration of medications into the skin or tumors. Therefore, the requirement for new approaches, comprising targeted and specialized medications, is more apparent than ever. Over the past few decades, nanomedicine has advanced therapeutic strategies by enhancing biodistribution, bioavailability, stability, pharmacokinetics, and targeted delivery, offering a promising alternative to current therapies [7–9].

Different approaches for drug delivery have been expressed to expand the effectiveness and minimize the side effects of topical therapeutic medications [10, 11]. The solid nanoscale fibers, known as nanofibers (NFs), possess sub-micrometric dimensions and desirable characteristics, including a substantial surface area per unit volume, significant porosity, applicable mechanical strength, and straightforward fabrication [12, 13]. Due to its simplicity of use and ability to regulate the features of NFs, electrospinning is the most frequently applied technique for NFs production [14, 15]. Polymers derived from natural sources, such as cellulose, chitosan (CS), collagen, starch, and silk, together with synthetic polymers like PVA, polycaprolactone (PCL), poly (lactic-co-glycolic acid), polyethylene oxide, and polyvinylpyrrolidone, are commonly applied to make electrospun NFs [16, 17]. Therefore, combined NFs with improved mechanical properties can be created by merging natural polymers with synthetic ones [13, 18]. One of the exciting features of NFs is their capacity to carry high loads of various therapeutic agents, including drugs, bioactive components, and plant extracts, thereby preventing their degradation and enabling controlled release to achieve maximum combined therapeutic effects [18, 19].

Indirubin is the main bioactive component of *Indigo naturalis*, a bis-indole alkaloid used in traditional Chinese medicine [20]. Numerous studies have divulged the potential therapeutic activities of indirubin, including its anti-inflammatory, antiviral, immunoregulatory, neuroprotective, and especially anticancer properties [21, 22]. Indirubin and its derivatives demonstrate significant anticancer properties, showing effectiveness against various malignant tumors, including leukemia, colorectal cancer, breast cancer, glioma, and melanoma [23–25]. These anticancer effects are primarily exerted by inhibiting tumor cell proliferation, invasion, migration, and angiogenesis [20–24, 26]. Despite these promising activities, several limitations affect their clinical usage and reduce their therapeutic effects, such as poor solubility in water, which leads to low absorption and bioavailability when administered orally, metabolic instability requiring frequent dosing or structural modifications, and high extraction and separation costs that result in low yields [20, 21, 23, 24, 27].

Hwa Seo et al. developed a mixed micellar formulation that could enhance the solubility and transdermal delivery of the poorly water-soluble indirubin analogue KY19382. The optimal surfactant-to-co-surfactant ratio improved solubility, stability, and skin permeation [28]. In another study, Wan-Ling Hsieh et al. developed a topical indigo naturalis ointment containing indirubin, which significantly down-regulates CDC25B expression at both mRNA and protein levels, thereby inhibiting epidermal keratinocyte proliferation by blocking EGFR activation and EGF-induced progression [29].

Our study aimed to develop nanofibrous scaffolds composed of PVA and CS, combined with indirubin, to investigate their cytotoxic effects on A375 melanoma cells. To the best of our knowledge, this research study has not yet been carried out. We inspected the properties of the obtained NFs, including physicochemical properties such as morphology, porosity, water absorption rate, and drug release profile. Besides, in vitro assays were conducted to assess the cytotoxic effects and determine the extent of apoptosis through flow cytometry analysis.

## Materials and methods

### Materials

Indirubin (MW: 262.26 g.mol^− 1^, CAS No. 479-41-4), PVA (MW = 72,000 Da, Hy = 99.5%) and chitosan(Mw: 50–190 KDa, 75–85% deacetylated) were purchased from Sigma-Aldrich (Germany). Acetic acid and ethanol were acquired from Merck Company (Darmstadt, Germany). Methanol and chloroform were procured from Mojallali Company (Iran). Cell culture reagents, including Dulbecco’s modified Eagle’s medium (DMEM) with high glucose, fetal bovine serum (FBS), 0.05% trypsin/EDTA, 3-(4,5-dimethylthiazol-2-yl-2,5-diphenyltetrazolium bromide) (MTT), and phosphate-buffered saline (PBS), were supplied by Gibco (Marcq-enBarœul, France). All other chemicals necessary for maintaining the integrity of the experiments were of standard analytical grade.

### Preparation of PVA/CS NFs loaded with indirubin

The PVA/CS NFs loaded with indirubin were prepared using Fanavaran Nano Meghyas’s electrospinning machine (Fanavaran Nano Meghyas Ltd., Co., Tehran, Iran). Initially, PVA/CS NFs were prepared to achieve the desired diameter and shape, after which the indirubin drug was loaded into the NFs. To find the optimal PVA/CS NFs, an 80:20 v/v mixed polymer blend was formulated using PVA (10% w/v in distilled water) and CS (4% w/v in 1% acetic acid) [30]. Then, indirubin, with concentrations of 5%, 10%, and 15% w/w, was added to the PVA/CS polymer solution and then subjected to the electrospinning process. The electrospinning procedure was conducted with a consistent flow rate of 1 mL/hr, an applied electric field strength of 18 kV, and a distance of 18 cm between the needle tip and the collector. Once the desired thickness was achieved, the fiber mats were cross-linked using glutaraldehyde vapor and subsequently detached from the aluminium sheet. The mats were then placed in a vacuum desiccator for 12 h at 25 °C to eliminate any remaining glutaraldehyde before being stored for further analysis and investigation.

### Diameter and morphology characterization

The diameter and surface morphology of the NFs were characterized through scanning electron microscopy (SEM), applying an accelerating voltage of 20.0 kV. The analysis was conducted using the HITACHI S-4700 instrument (Japan). Prior to imaging, the dried scaffolds—comprising free, 5%, 10%, and 15% weight fractions—were subjected to gold sputter coating. Additionally, the average diameter of the fibers was determined using ImageJ software by measuring the diameters of 100 randomly selected fibers in each microscopic image.

### FTIR spectroscopy

FTIR spectroscopy was employed to examine both the indirubin loading and the interactions between the fiber ingredients. A Shimadzu IRAffinity-1 S spectrophotometer was exploited for the spectroscopic analysis, with measurements spanning from 400 to 4000 cm^− 1^ at a temperature of 25 °C.

### Evaluations of porosity

The liquid displacement technique was employed to determine the porosity of both the loaded and free PVA/CS scaffolds with indirubin [31]. The scaffolds were submerged in pure ethanol for 1 h, after which the porosity was calculated using Eq. (1):

1 $$\:\mathrm{P}\mathrm{o}\mathrm{r}\mathrm{o}\mathrm{s}\mathrm{i}\mathrm{t}\mathrm{y}\:\left(\mathrm{\%}\right)\:=\:\left[\right(\mathrm{V}1\:-\:\mathrm{V}2)/(\mathrm{V}2\:-\:\mathrm{V}3\left)\right]\:\times\:\:100$$

*V*_*1*_ indicates the initial volume of ethanol, *V*_*2*_ denotes the volume after immersion, and *V*_*3*_ represents the volume of ethanol following the removal of the mat after one hour.

### Evaluations of liquid absorption

To determine the liquid absorption capacity of the prepared scaffolds, initially, the dry weight of the scaffolds was precisely recorded. The scaffolds were then immersed in a PBS solution at pH 7.4 and maintained at room temperature. After scheduled time intervals, the samples were removed from the solution and placed onto filter paper to remove excess liquid. Subsequently, the wet weight of the scaffolds was recorded [31]. The liquid absorption percentage of the scaffolds (W) was calculated using Eq. (2):

2 $$\:\mathrm{W}\:\left(\mathrm{\%}\right)\:=\:\left[\right(\mathrm{W}2-\mathrm{W}1)\:/\:\mathrm{W}1]\:\times\:\:100$$

*W*_*1*_ represents the dry weights of the scaffolds. *W*_*2*_ represents the wet weights of the scaffolds.

### Evaluations of wettability

The wettability characteristics of the scaffolds were evaluated using the sessile drop method, where the contact angle was measured using a static optical contact angle measurement setup (CAG-10, Jikan, Iran). A 5 µL drop of distilled water was carefully placed on the surface of each sample, and images of the water droplet were captured at room temperature. The angles were then measured.

### Mechanical properties

The mechanical properties of the prepared scaffolds were evaluated using a universal testing machine (Santam, Iran). For testing, the scaffolds were cut into pieces with dimensions of 3 cm × 0.5 cm. They were then securely placed in the grips of the machine and subjected to tensile stress at a rate of 10 mm/min, using a 10 N load cell to measure the applied force [32–35]. Subsequently, the stress-strain curves were plotted, and the mechanical properties of the samples were determined.

### Antioxidant assay

The antioxidant activity of specimens, including free indirubin, free PVA/CS NFs, and indirubin-loaded NFs (Non-cross-linked scaffold containing an equivalent amount of free indirubin), was evaluated using the DPPH radical scavenging assay. Therefore, samples in various concentrations were mixed in a 10^− 4^ M DPPH ethanol solution. Also, ascorbic acid was used as the standard antioxidant at the same concentrations. They were incubated in a dark ambient for 60 min at room temperature. After the end of the incubation period, absorbance measurements of the solution were taken at 517 nm with a microplate reader (PerkinElmer, USA) [36], and the percentage of antioxidant activity was calculated using Eq. (3):

3 $$\begin{aligned}&\mathrm{A}\mathrm{n}\mathrm{t}\mathrm{i}\mathrm{o}\mathrm{x}\mathrm{i}\mathrm{d}\mathrm{a}\mathrm{n}\mathrm{t}\:\mathrm{a}\mathrm{c}\mathrm{t}\mathrm{i}\mathrm{v}\mathrm{i}\mathrm{t}\mathrm{y}\:\left(\mathrm{\%}\right)\cr & \quad=\:\left[\right(\mathrm{A}\mathrm{c}\mathrm{o}\mathrm{n}\mathrm{t}\mathrm{r}\mathrm{o}\mathrm{l}\:-\:\mathrm{A}\mathrm{s}\mathrm{a}\mathrm{m}\mathrm{p}\mathrm{l}\mathrm{e})/\:\mathrm{A}\mathrm{c}\mathrm{o}\mathrm{n}\mathrm{t}\mathrm{r}\mathrm{o}\mathrm{l}]\:\times\:\:100\end{aligned}$$

A_control_ and A_sample_ are the absorbance of DPPH in the absence and presence of samples, respectively.

### In vitro drug release

The membrane diffusion method, utilizing a dialysis membrane, was employed to determine the release profile of encapsulated indirubin from the prepared 10 wt% indirubin nanofibrous scaffolds. Indirubin-loaded NFs were placed in a dialysis bag (cut-off 12000 Da) and immersed in a defined volume of PBS with 10% methanol at 37 ± 0.5 °C and pH 7.4 [37]. One mL of the medium was collected at specific time intervals, then to maintain sink conditions, an equal volume of fresh medium was substituted. The concentration of indirubin in the collected samples was quantified using a spectrophotometer at 290 nm, based on a plotted standard curve [38], which enabled the calculation of cumulative release at each interval. A release profile curve was plotted to illustrate the cumulative amount of indirubin released over time.

### Cytotoxicity assay

The potential cytotoxicity effect of samples was evaluated using a direct MTT colorimetric assay against A375 human melanoma cells, provided by the Iranian Biological Resource Center. As follows, 10,000 A375 cells were seeded into each well of a 96-well plate in DMEM medium supplemented with 10% FBS, and the plate was incubated at 37 °C with 5% CO_2_. After 24 h, the cells were exposed to UV-sterilized scaffolds, including free PVA/CS NFs and 5–15 w/w% indirubin-loaded NFs, as well as free indirubin (50–150 µg per well, equivalent to the loaded values in the NFs), and incubated for 24 and 48 h. After each incubation time, the cells were washed with PBS (pH 7.4) and subsequently treated with an MTT solution (5 mg/mL) for three hours. Afterwards, the MTT solution was discarded, and formazan crystals were solubilized in 100 µL of dimethyl sulfoxide. Absorbance was recorded using the ELISA plate reader at 570 nm, and cytotoxicity was determined using Eq. (4) [39].

4 $$\begin{aligned}&\mathrm{C}\mathrm{y}\mathrm{t}\mathrm{o}\mathrm{t}\mathrm{o}\mathrm{x}\mathrm{i}\mathrm{c}\mathrm{i}\mathrm{t}\mathrm{y}(\mathrm{\%})\cr & \quad=\frac{(\mathrm{A}\mathrm{b}\mathrm{s}\mathrm{o}\mathrm{r}\mathrm{b}\mathrm{a}\mathrm{n}\mathrm{c}\mathrm{e}\:\mathrm{o}\mathrm{f}\:\mathrm{c}\mathrm{o}\mathrm{n}\mathrm{t}\mathrm{r}\mathrm{o}\mathrm{l}-\mathrm{A}\mathrm{b}\mathrm{s}\mathrm{o}\mathrm{r}\mathrm{b}\mathrm{a}\mathrm{n}\mathrm{c}\mathrm{e}\:\mathrm{o}\mathrm{f}\:\mathrm{s}\mathrm{a}\mathrm{m}\mathrm{p}\mathrm{l}\mathrm{e})}{(\mathrm{A}\mathrm{b}\mathrm{s}\mathrm{o}\mathrm{r}\mathrm{b}\mathrm{a}\mathrm{n}\mathrm{c}\mathrm{e}\:\mathrm{o}\mathrm{f}\:\mathrm{c}\mathrm{o}\mathrm{n}\mathrm{t}\mathrm{r}\mathrm{o}\mathrm{l})}\cr & \quad\times\:100\end{aligned}$$

### Measuring the cell apoptosis

The induction of apoptosis was quantified by flow cytometry analysis of Annexin V-FITC and propidium iodide (PI) staining (Apoptosis Detection Kit, Kalazist, Iran) of A375 skin melanoma cells. The melanoma cells (3 × 10^5^) were cultured in each 12-well plate and treated with the samples, including UV-sterilized scaffolds (5–15 w/w% indirubin-loaded NFs), as well as free indirubin (50–150 µg per well, equivalent to the loaded values in the scaffolds) for 24 h. After incubation, they were rinsed with PBS, trypsinized, and then subjected to centrifugation at 800 rpm for 3 min. The cells were subsequently resuspended in PBS and combined with binding buffer, then incubated with Annexin V-FITC and PI dyes for 20 min. Finally, the stained cells were analyzed by flow cytometry using the Calibur BDFACS instrument (Attune NxT, Thermo Fisher Scientific Co., Ltd.).

### Analytical statistics

The data was evaluated using GraphPad Prism 9. ImageJ was used to measure the diameter distribution of the obtained nanoscaffolds. One/two-way ANOVA and t-tests were used to examine variations between groups. Variations with a p-value below 0.05 were considered statistically significant, labeled as ‘ns’ for *p* > 0.05 (not significant), * for *p* < 0.05, ** for *p* < 0.01, *** for *p* < 0.001, and **** for *p* < 0.0001.

## Results and discussion

### Scaffolds’ characteristics of morphology and diameter

**Fig. 1:**
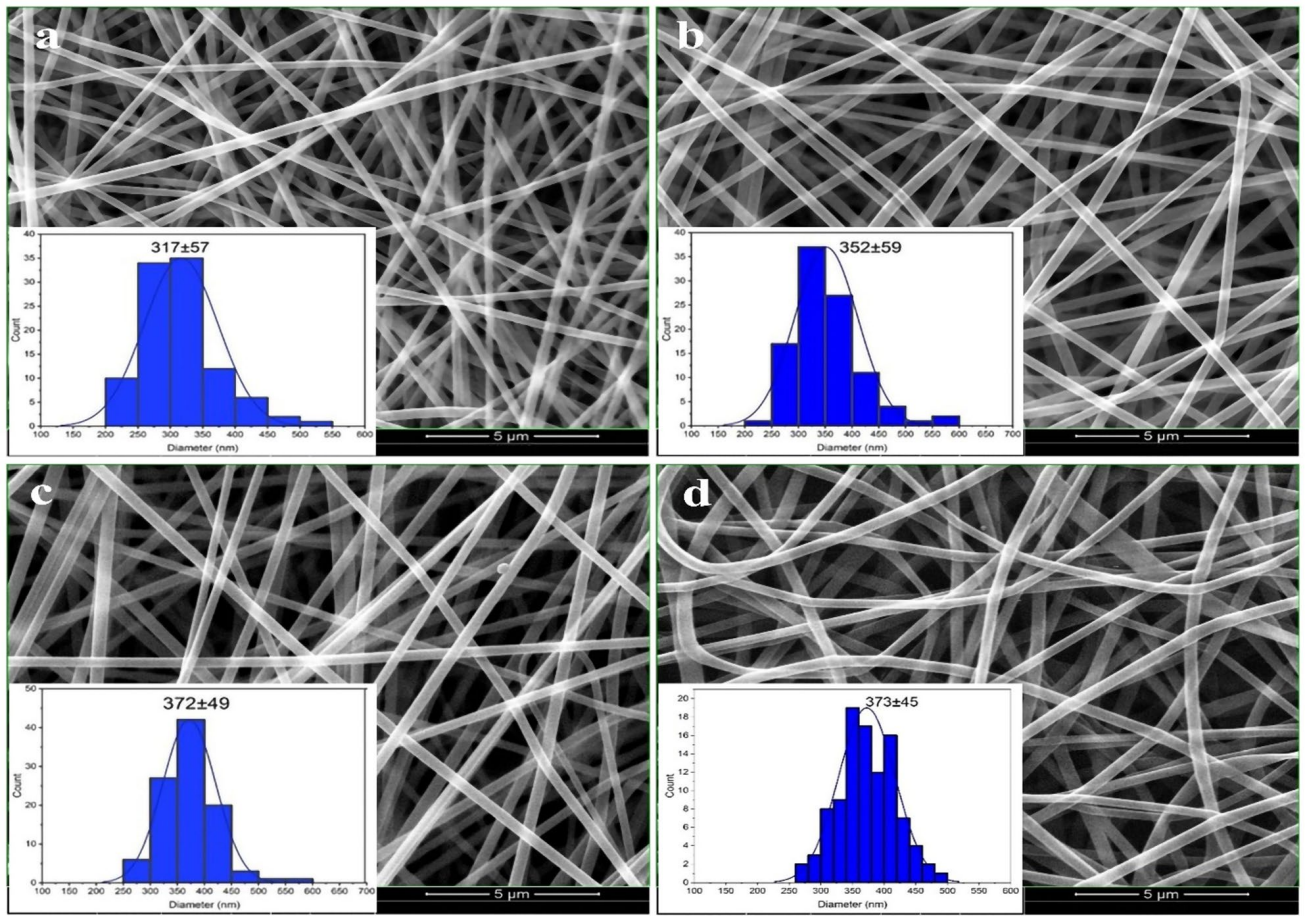
SEM micrograph of **a**) free PVA/CS NFs, **b**) 5 wt% indirubin-loaded PVA/CS NFs, **c**) 10 wt% indirubin-loaded PVA/CS NFs, and **d**) 15 wt% indirubin-loaded PVA/CS NFs. Scale bars = 5 μm and magnification = 8000 X

Figure 1 displays the SEM images and size distribution histograms of four types of scaffolds. The free PVA/CS scaffold exhibited a consistent morphological feature, including a uniform diameter, sleek surface, absence of beads, and a porous structure. As observed in Fig. 1a, the mean diameter of PVA/CS NFs is 317 ± 57 nm. 5 wt% of indirubin provided the NFs with a consistent structure and an average diameter of 352 ± 59 nm (Fig. 1b). Furthermore, the SEM image revealed that incorporating 10 wt% of indirubin resulted in NFs that exhibited a consistent shape, with a diameter distribution of 372 ± 49 nm (Fig. 1c). The 15 wt% of indirubin augmented the average diameter of the NFs to 373 ± 45 nm (Fig. 1d). Although increasing the concentration of indirubin to 15 wt% resulted in changes in the size distribution of the fibers, this increase was not considerable. The nanoscale diameter of fibrous scaffolds provides a high surface-to-volume ratio, closely resembling the extracellular matrix (ECM), which enhances cell adhesion and proliferation through increased interaction between cells and the scaffold material [40, 41]. CS plays a crucial role in improving the electrospinning performance and functional properties of PVA-based nanofibers. Due to its polycationic nature, CS enhances the electrical conductivity of the spinning solution, promoting stable jet formation and facilitating the generation of uniform fibers. In addition, the high molecular weight and viscosity of CS act as a thickening agent, improving solution spinnability and reducing the formation of beads during fiber deposition [38, 42, 43]. The presence of amino and hydroxyl groups in CS also promotes hydrogen bonding with PVA chains, resulting in a homogeneous polymeric blend with enhanced mechanical integrity and morphological uniformity. In this study, incorporating CS not only improved the electrospinnability of the PVA matrix but also endowed the resulting nanofibers with favorable biocompatibility, hydrophilicity, and structural similarity to natural extracellular matrices—features essential for the localized drug delivery and tissue-interaction performance of indirubin-loaded scaffolds [44–46].

### FTIR studies

**Fig. 2:**
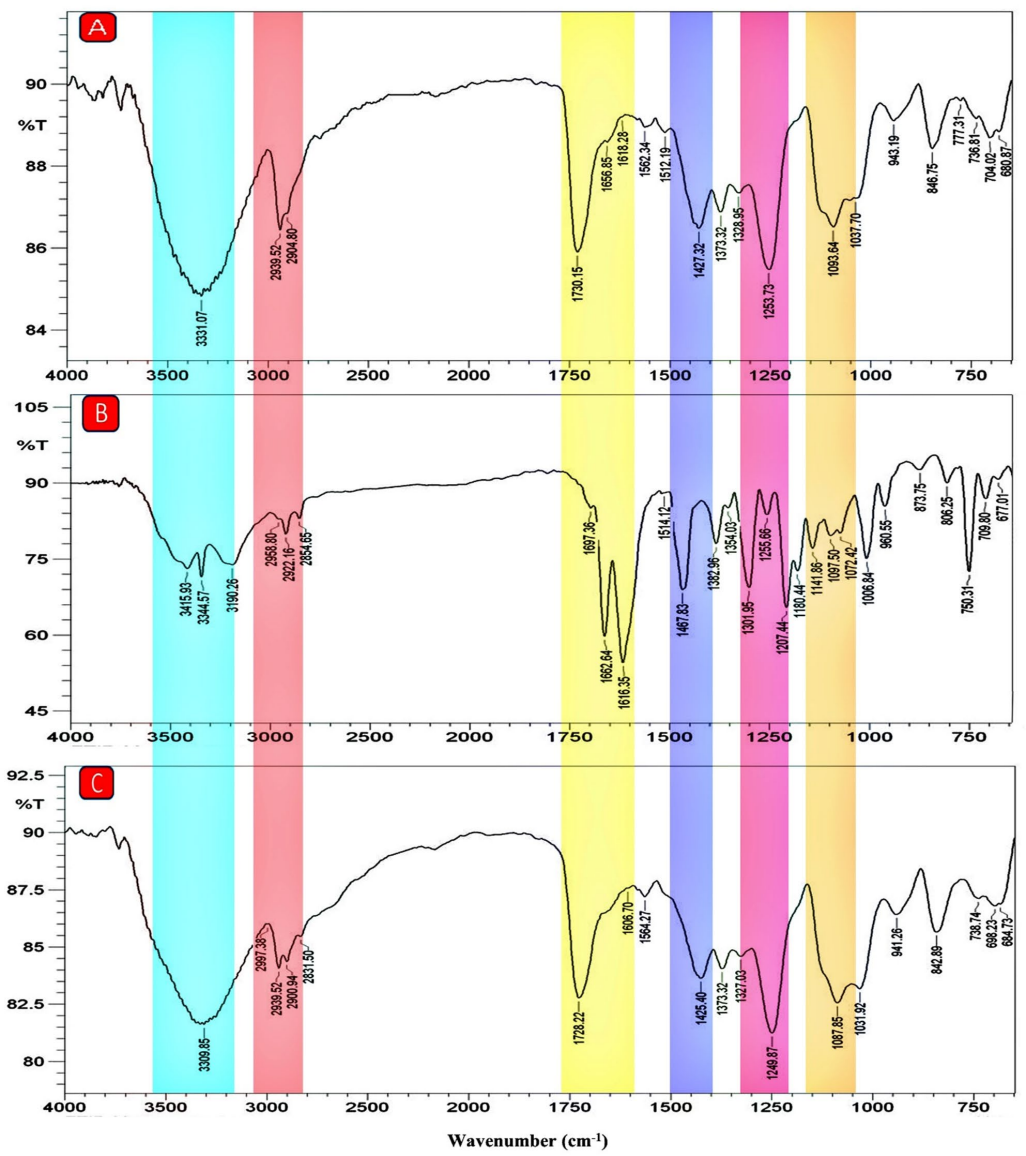
FTIR spectrum of synthetic free PVA/CS NFs (**A**), free indirubin (**B**), and PVA/CS NFs loaded with indirubin

FTIR spectroscopy was used to analyze the functional groups and molecular interactions, and to confirm the incorporation of indirubin within the PVA/CS nanofibrous scaffolds. As presented in Fig. 2, the FTIR spectrum of the PVA/CS scaffold (Fig. 2A) exhibited characteristic peaks at 1093 cm⁻¹ and 1253 cm⁻¹ corresponding to C–O–C stretching vibrations, and a peak at 1730 cm⁻¹ related to C = O stretching from residual vinyl acetate groups of PVA and acetyl groups in chitosan. The -CH_2_ group induces both asymmetrical and symmetrical elongation vibrations, contributing to the absorption patterns at 2904 cm^− 1^ and 2939 cm^− 1^. Additionally, the broad absorption band centered at 3331 cm⁻¹ is attributed to overlapping O–H and N–H stretching vibrations from hydroxyl and amino groups of PVA and CS, respectively [31, 47].

The FTIR spectrum of free indirubin (Fig. 2B) displayed a broad band in the range of 3344–3415 cm⁻¹ assigned to N–H stretching vibration. The C–H stretching of aromatic rings appeared between 2854 and 2958 cm⁻¹, while bands observed in the region of 1514–1697 cm⁻¹ correspond to C = C, C = O, and N–H stretching. Additional peaks at 1467 cm⁻¹ and 1301 cm⁻¹ were attributed to C–C and C–N stretching vibrations, respectively (Fig. 2B) [48].

The FTIR spectrum of indirubin-loaded PVA/CS scaffolds (Fig. 2C) combined characteristic peaks from both indirubin and the polymeric components. The broad and intense band near 3309 cm⁻¹ indicates intermolecular hydrogen bonding between the N–H groups of indirubin (or amide groups of CS) and O–H groups of PVA [49]. The aromatic C–H stretching vibration was observed in the range of 2831–2939 cm⁻¹. A distinct peak at 1728 cm⁻¹ corresponds to C = O stretching, confirming the presence of indirubin alongside residual acetyl groups from PVA and CS [50, 51]. Furthermore, the peaks at 1606 cm⁻¹, 1564 cm⁻¹, and 1249 cm⁻¹ were attributed to C = C, N–H, and C–O stretching vibrations, respectively [48]. Minor peak shifts and overlapping bands were detected, indicating physical interactions and successful incorporation of indirubin into the PVA/CS matrix [31].

### Water absorption results

**Fig. 3:**
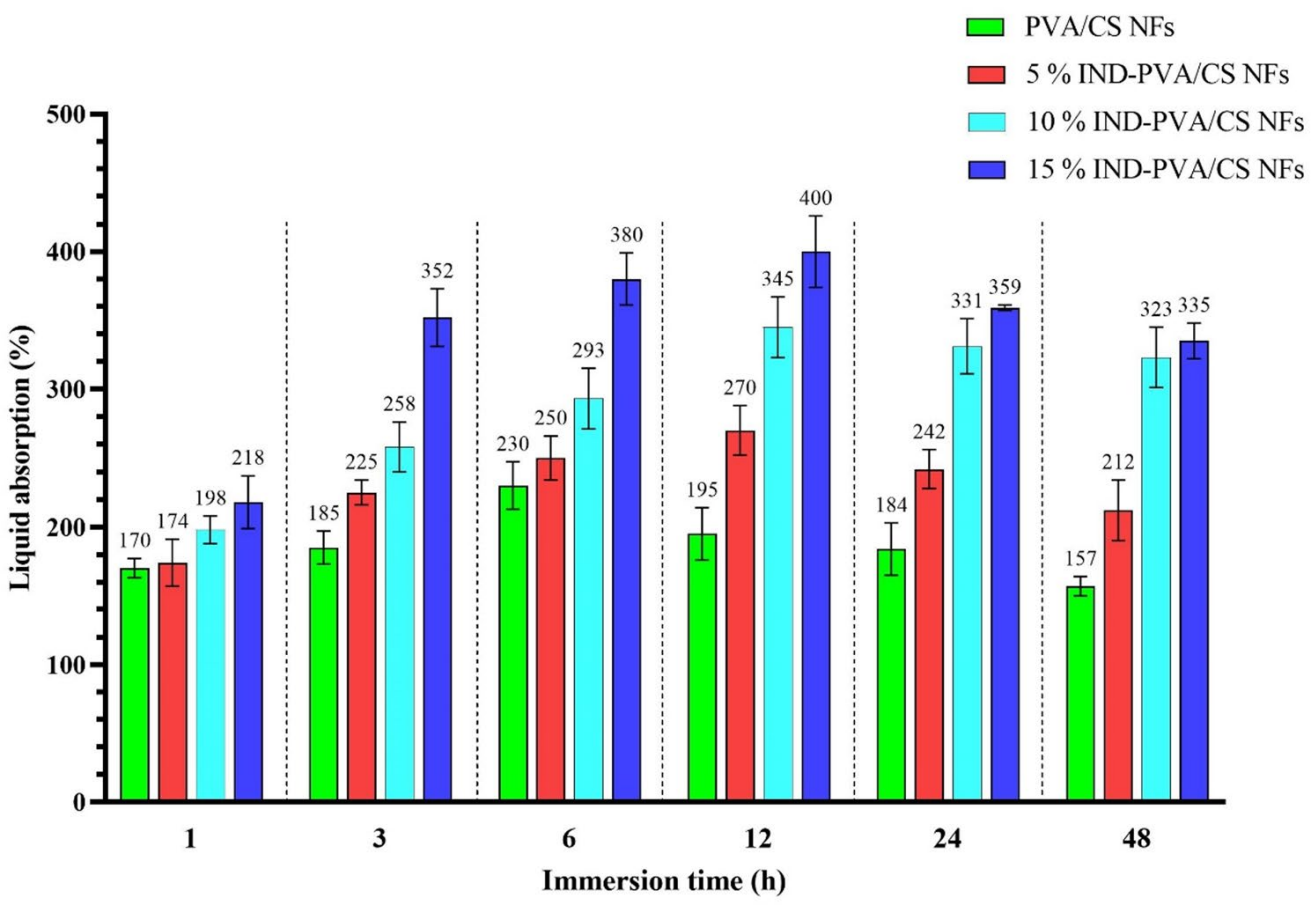
The capacity to absorb water of free PVA/CS and 5, 10, and 15 wt% indirubin-loaded PVA/CS NFs

As illustrated in Fig. 3, the water absorption capacities of the scaffolds were measured using the immersion method. After 1 h of immersion, the free PVA/ CS scaffold, 5 wt% indirubin, 10 wt% indirubin, and 15 wt% indirubin loaded scaffold exhibited an average water absorption percentage of 170 ± 7%, 174 ± 17%, 198 ± 10% and 218 ± 29% respectively. After 48 h of immersion, the water uptake percentages increased to around 157 ± 8%, 212 ± 28%, 323 ± 15%, and 335 ± 10% for the obtained nanoscaffolds, respectively. These findings suggest that the scaffolds have a rapid water absorption rate, likely due to the higher porosity of the loaded scaffolds and the presence of hydrophilic functional groups, such as hydroxyl and amino groups, in the CS and PVA chains [52]. Indirubin PVA/CS scaffold’s water absorption capacity was greater than that of free PVA/CS NFs. It seems that the high swelling ratio in the indirubin-loaded PVA/CS NFs is attributed to the formation of an enhanced flexible network due to intra-polymer chain reactions, improved flexibility, and further hydrophilic structures [53, 54]. The presence of hydroxyl groups in PVA contributes to its water absorption capacity because cellulose/PVA films exhibited higher water absorption (73%–78%) than cellulosic films (33%–42%) [55]. Electrospun NFs can efficiently load drugs and respond to specific conditions for controlled release due to their high specific surface area and porosity [56]. In the context of localized melanoma therapy, the scaffolds’ water absorption capacity is a key functional property that enables controlled drug release, rapidly absorbs interfacial water within seconds, and establishes physical adhesion with the skin, maintains a hydrated microenvironment at the tumor site to support cellular activities and tissue regeneration, and promotes scaffold–tissue interactions [57, 58].

### Porosity results

**Fig. 4:**
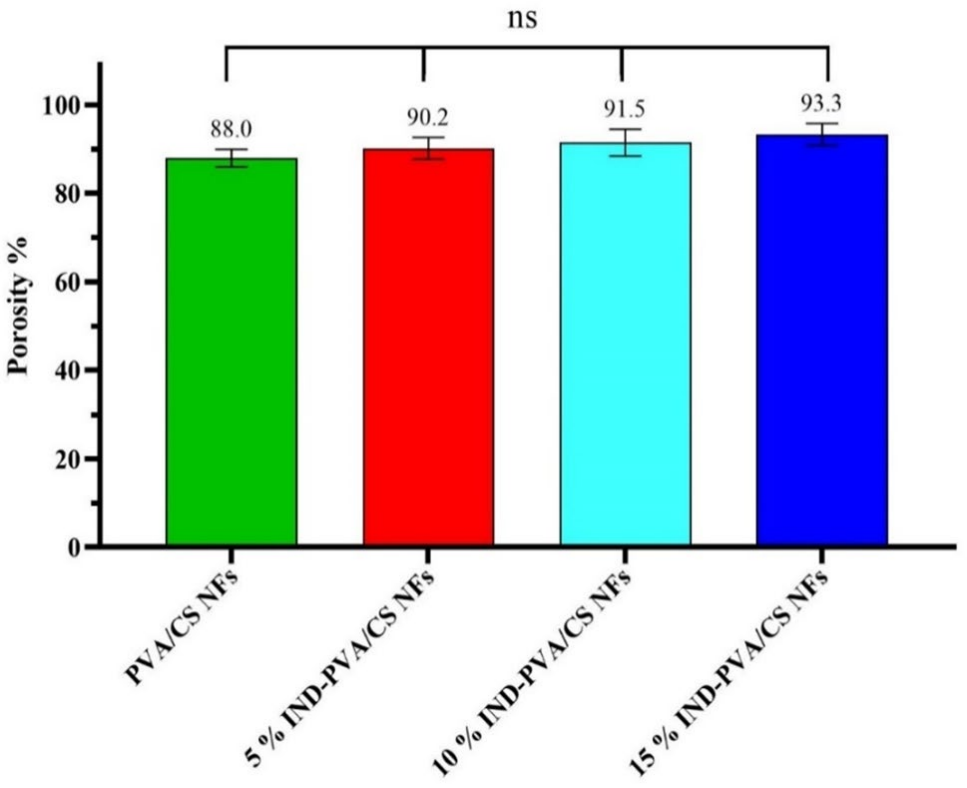
Porosity diagram for fabricated NFs. (Above the columns, the average values are presented; ns represents *p* > 0.05, and **** denotes *p* < 0.05)

The scaffold’s porosity is crucial for facilitating cell proliferation on the scaffold and its subsequent degradation [59, 60]. Porosity analysis indicated that the PVA/CS scaffold without loading (free PVA/CS) had a porosity of 88% of the total scaffold volume. At the same time, 5 wt%, 10 wt%, and 15 wt% indirubin-loaded NFs exhibited a slightly higher porosity of 90.2%, 91.5%, and 93.3%, respectively (Fig. 4). While there was a rise in porosity with indirubin loading, this alteration was not statistically meaningful (*p* > 0.05) [61]. The porosity of the NFs typically ranges from 75% to 82% in PVA/CS scaffolds [62]. These highly porous structures facilitate nutrient exchange and cell attachment, making them suitable for skin regeneration [63, 64]. The results suggest that the electrospun NFs provide numerous pores, allowing for the efficient flow of nutrients and gases, as well as absorbing fluids [60, 65]. The observed slight increase in scaffold porosity with higher indirubin loading, despite relatively uniform fiber morphologies seen in SEM images. They explain that the minor porosity changes can be attributed to alterations in the physicochemical properties of the polymer solution during electrospinning, such as viscosity, conductivity, and polymer-drug interactions, which may subtly affect fiber packing density and inter-fiber spacing.

### Wettability results

**Fig. 5:**
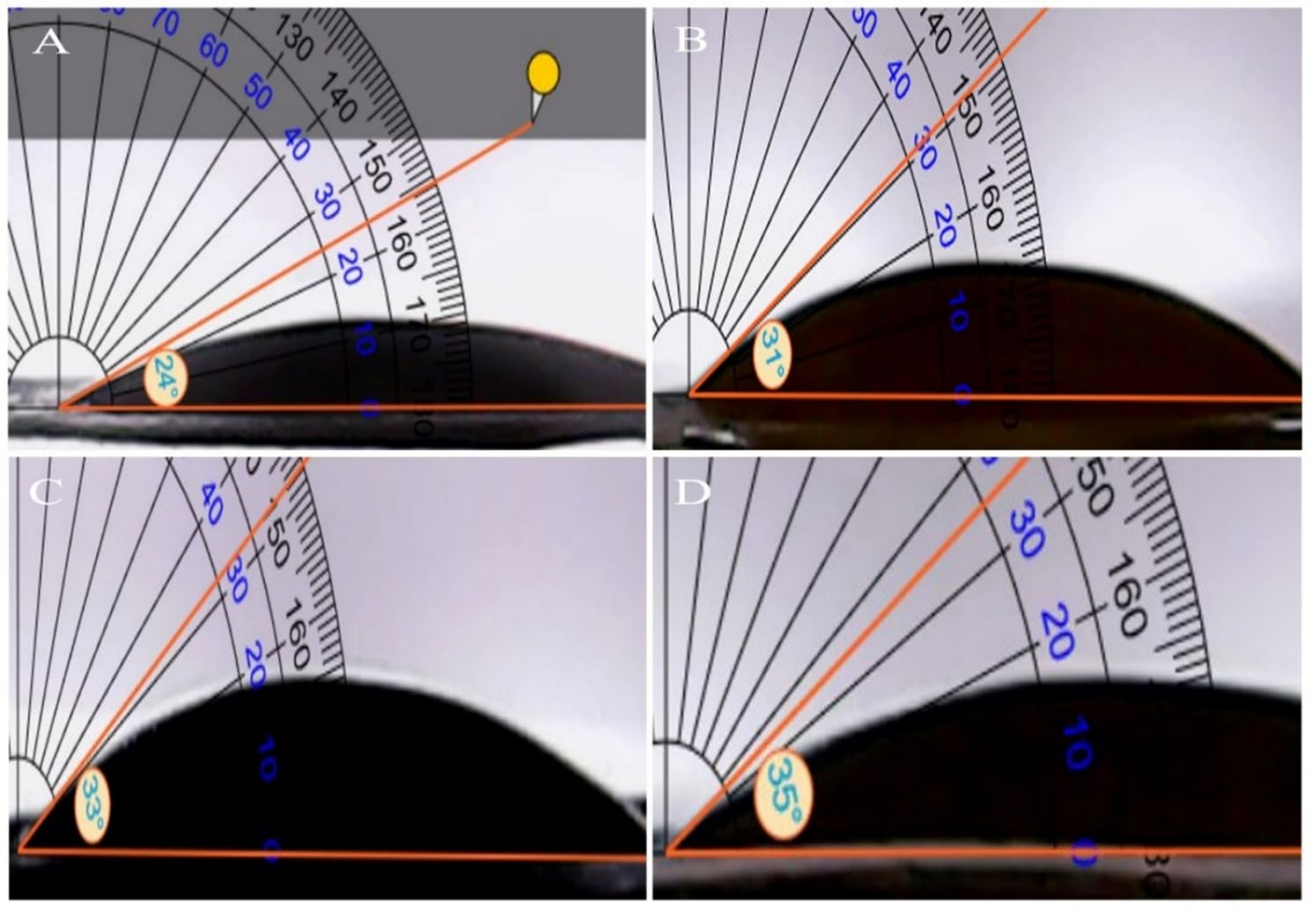
The water contact angle for fabricated NFs. **A**) free PVA/CS NFs, **B**) 5 wt% indirubin-loaded PVA/CS NFs, **C**) 10 wt% indirubin-loaded PVA/CS NFs, and **D**) 15 wt% indirubin-loaded PVA/CS NFs

The water contact angle was used to assess the hydrophilicity of the scaffold surface. This feature may impact the maintenance of moisture levels and the adhesion and proliferation of cells on the nanoscaffolds [59, 66]. Therefore, the hydrophilicity of the scaffolds was quantified. Results showed that the free PVA/CS NFs had a contact angle of around 24°, indicating a highly hydrophilic and wettable surface. In comparison, incorporating indirubin at concentrations of 5, 10, and 15 wt% resulted in a slight increase in contact angles, measured at approximately 31°, 33°, and 35°, respectively (Fig. 5). The results showed a decrease in surface hydrophilicity and wettability with increasing indirubin concentration. However, all formulations maintained relatively low contact angles, indicating overall hydrophilic properties that are beneficial for potential in vivo applications, such as in drug delivery and tissue regeneration [67, 68]. Hydrophilicity governs scaffold hydration and drug release. In localized melanoma treatment, a moderately hydrophilic surface supports effective drug delivery and a favorable interaction with the lesion [69, 70].

### Mechanical results

**Fig. 6:**
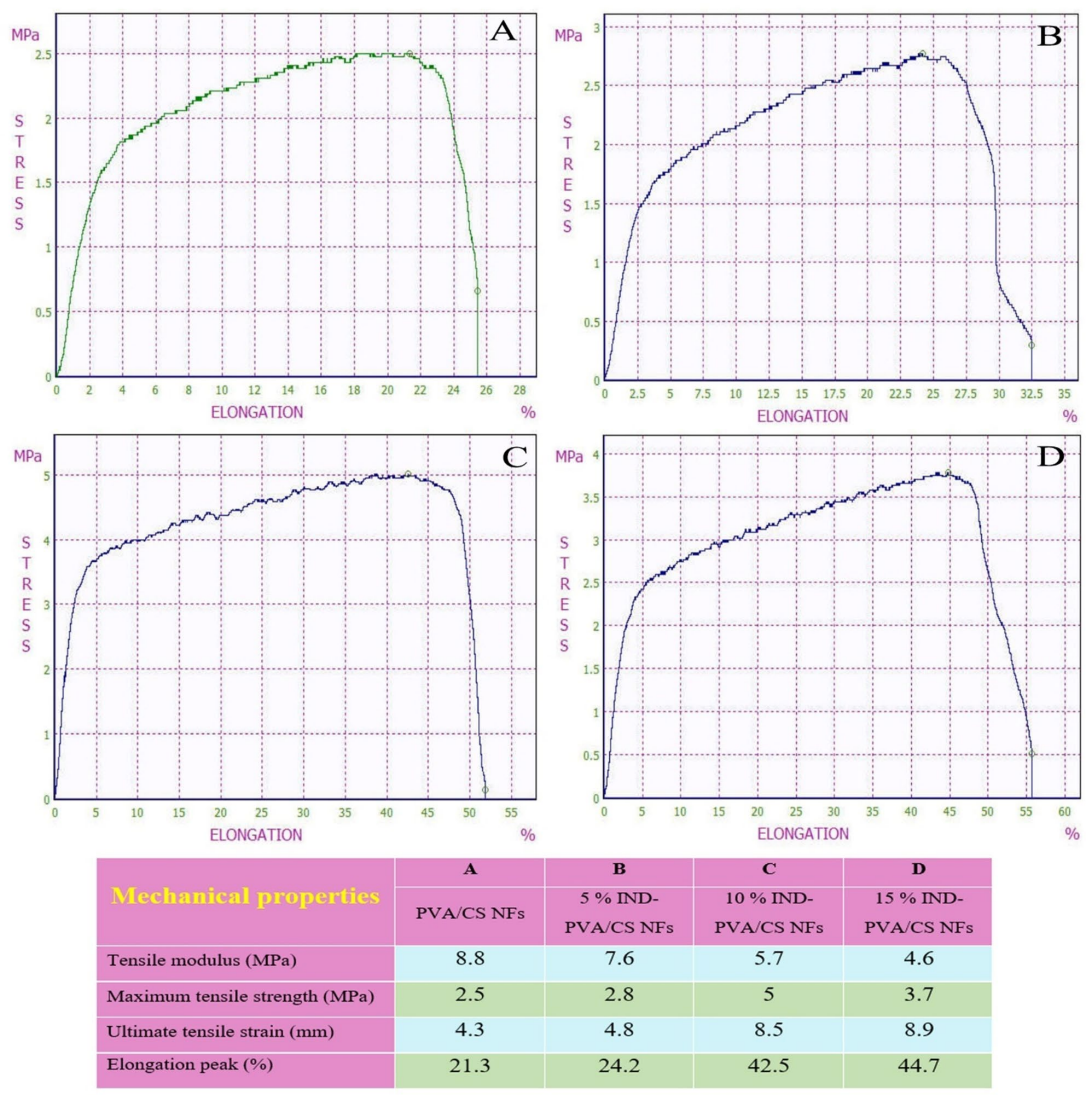
Strain stress diagram for **A**) free PVA/CS NFs, **B**) 5 wt% indirubin-loaded PVA/CS NFs, **C**) 10 wt% indirubin-loaded PVA/CS NFs, and **D**) 15 wt% indirubin-loaded PVA/CS NFs

The scaffolds should possess mechanical properties, particularly tensile strength, that are sufficient to maintain structural integrity throughout use [71]. Figure 6 displays the stress-strain curves of the scaffolds. The PVA/CS scaffold exhibited an average Young’s modulus of 8.8 MPa, a maximum tensile strength of 2.5 MPa, and an ultimate tensile strain of 21.3%. Electrospun NFs prepared from the PVA polymer exhibited notable mechanical properties and chemical resistance, along with a high swelling ability [72]. In comparison, the PVA/CS scaffold loaded with 5 wt% indirubin showed a Young’s modulus of 7.6 MPa, a maximum tensile strength of 2.8 MPa, and an ultimate tensile strain of 24.2%. The PVA/CS scaffold loaded with 10 wt% indirubin demonstrated a Young’s modulus of 5.7 MPa, a maximum tensile strength of 5 MPa, and an ultimate tensile strain of 42.5%. The higher tensile strength observed at 10 wt% loading likely results from an optimal level of physical intermolecular interactions between indirubin molecules and the PVA/CS chains, which reinforces the polymeric network. Finally, the 15 wt% indirubin-loaded PVA/CS scaffold had a Young’s modulus of 4.6 MPa, a maximum tensile strength of 3.7 MPa, and an ultimate tensile strain of 46.7%. The enhancement in mechanical properties can be attributed to the formation of an additional bonding network between indirubin and the electrospun PVA/CS NFs through physical interactions, such as hydrogen bonding [73].

### Antioxidant activity

**Fig. 7:**
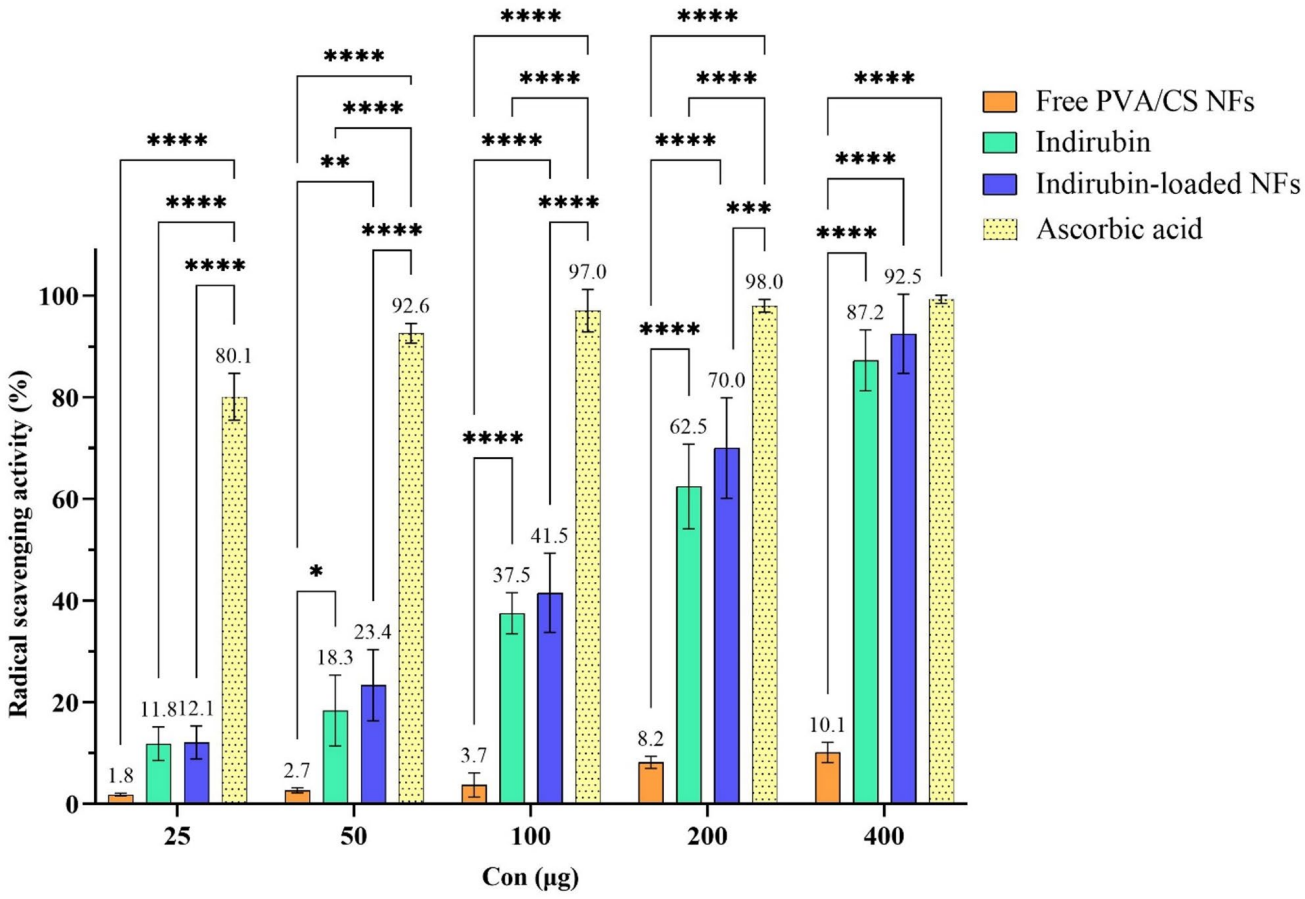
Antioxidant activities of samples at different concentrations confirmed by DPPH scavenging assay (The mean values are presented above the columns, (*p < 0.01, **p < 0.01, ****p < 0.0001))

Chronic inflammation drives melanoma progression as inflammatory cells release cytokines, chemokines, and reactive oxygen species (ROS) that promote proliferation, angiogenesis, and metastasis. Moderate ROS also activates cancer-related signaling pathways that support melanoma cell survival and growth [74–76]. The antioxidant activities of samples are revealed in Fig. 7. The free PVA/CS NFs showed negligible radical scavenging activity (less than 11% even at the highest dose), confirming that the nanofiber matrix itself is not the source of the antioxidant effect. The radical scavenging activity of free indirubin was compared with that of free PVA/CS NFs and indirubin-loaded NFs. At lower concentrations (25 and 50 µg), indirubin proved to be slightly more effective than free NFs alone, but that difference was not statistically significant. At higher concentrations (100, 200, and 400 µg), indirubin proved to be statistically significantly more active (*p* < 0.0001). Similarly, indirubin-loaded NFs showed no significant differences in activity compared to free indirubin at low concentrations; however, at higher doses, there was a marked difference in terms of radical scavenging activity as compared to free Indirubin. This enhancement was most pronounced at the highest concentration (400 µg), where indirubin-loaded NFs achieved 92.5% scavenging activity—significantly higher than free indirubin (87.2%) and approaching that of ascorbic acid (~ 100%). The reason for this could be the high surface-to-volume ratio and porosity of the scaffolds, which enhance the greater exposure and interaction of free DPPH radicals with the surface of the scaffolds and the loaded indirubin [77, 78]. Moreover, at low concentrations, the nanofiber formulation enhances the antioxidant activity of indirubin, although Ascorbic acid remains superior. At high concentrations, nanofiber delivery significantly boosts activity, bringing indirubin’s performance close to that of ascorbic acid and highlighting the advantage of the delivery system in improving bioavailability and radical scavenging efficiency. These findings suggest that the antioxidant effect of indirubin can be further enhanced by loading it into nanofibrous scaffolds, indicating a potent defense against oxidative stress–mediated damage, which may support melanoma treatment.

### In vitro drug release

**Fig. 8:**
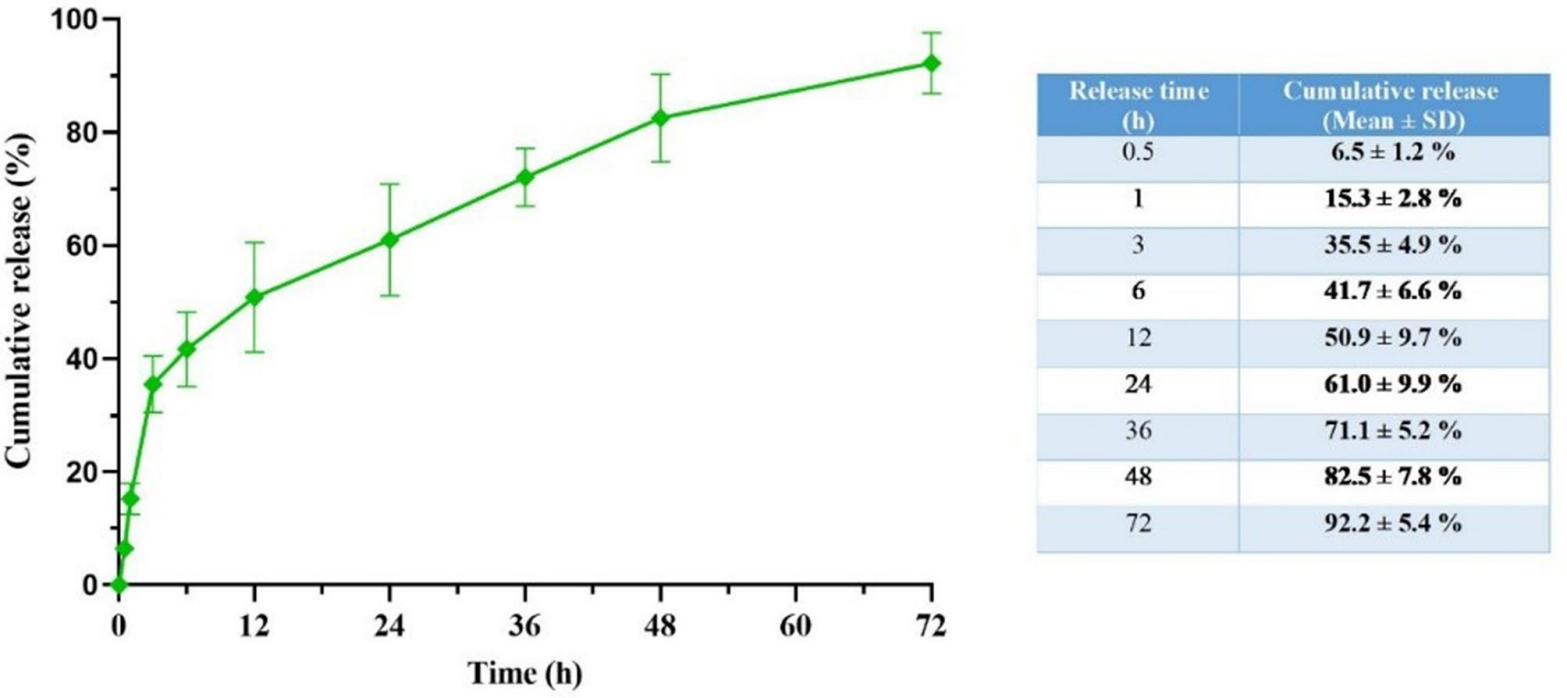
Cumulative release percentage of indirubin released from indirubin-loaded PVA/CS scaffold

Figure 8 illustrates the release pattern of indirubin from indirubin-loaded PVA/CS NFs in a laboratory setting throughout 72 h. After 6 h, approximately 41.7% ± 6.6% of indirubin was released. After 24 h, the released quantity increased to 61.0% ± 9.9%. Following a 72-h period, about 92.2% ± 5.4% of indirubin was released from PVA/CS NFs loaded with indirubin. The pattern of release of indirubin from indirubin-loaded PVA/CS NFs indicated rapid release during the first several hours. The fast release of the NFs can likely be attributed to the polymer matrix. After the initial burst phase, a slower release, similar to the delay phase, was observed. At this point, polymer degradation has a significantly greater impact on the release process than NF degradation. As a result, the drug release behavior from the NFs exhibited that indirubin was steadily and continuously released from the blended polymer over 72 h. The obtained electrospun NFs, with their constant and sustained release of the loaded drug, can offer lasting effects and reduce the consumption of high doses of medication required to hinder and eliminate cancerous cells effectively [19, 79]. The drug release profile from electrospun PVA/CS NFs, which typically completes almost entirely by 72 h, has significant implications for melanoma therapy [2]. Although this timeframe is relatively short compared to sustained-release systems designed to cover over several days, it may still hold clinical relevance, especially for rapid and localized drug delivery intended to attain high early cytotoxic concentrations at the tumor site [2]. Therefore, the 72-hour release from electrospun PVA/CS NFs could be clinically beneficial in combination therapies or specific treatment situations that need quick drug action, although tailoring of release duration might be required based on the therapeutic objective and drug used [80].

### In vitro cytotoxicity studies

**Fig. 9:**
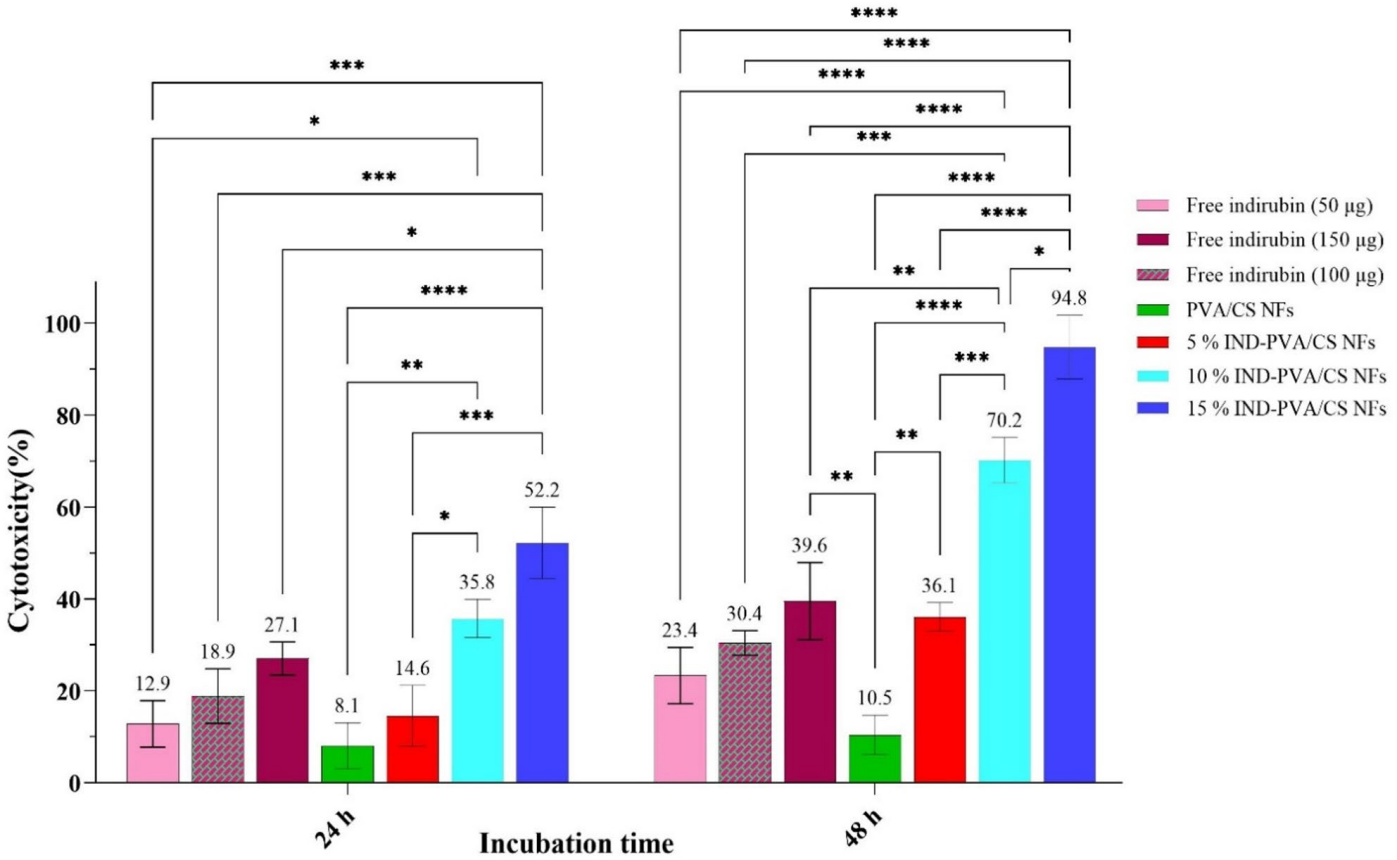
Cytotoxicity chart showing the response of A375 melanoma cells to different formulations over periods of 24 and 48 h (Non-significant differences are not shown, *p < 0.01, **p < 0.01, ****p < 0.0001)

Nanofibrous scaffolds can serve as a promising implantable platform for localized cancer treatment, offering tunable features such as a high surface area, high drug loading capacity, adjustable pore sizes, and sustained release capabilities [81, 82]. Therefore, the cytotoxic efficacy of indirubin-loaded nanofibrous scaffolds was evaluated against A375 melanoma cells and compared to an equivalent dose of the free drug. The cytotoxicity of treatments against A375 cells was about 12.9%, 18.9%, 27.1%, 8.1%, 14.6%, 35.8%, and 52.2%, after 24 h of incubation, and after 48 h of incubation was 23.4%, 30.4%, 39.6%, 10.5%, 36.1%, 70.2%, 94.8%, for free indirubin drug at concentrations of 50 µg, 100 µg, 150 µg, free PVA/CS NFs, 5% indirubin-PVA/CS NFs, 10% indirubin-PVA/CS NFs, and 15% indirubin-PVA/CS NFs, respectively (Fig. 9). Cell viability obviously decreased with increasing exposure time to free compounds and NFs from 24 to 48 h, indicating increased toxicity. Remarkable cytotoxicity was not observed in the free PVA/CS scaffold, reaching a maximum of about 10% after 48 h of incubation, indicating the safety and biocompatibility of the prepared NFs [62, 83]. While the loaded scaffolds, especially those with 15 wt% indirubin, exhibited the highest death rate in A375 melanoma cancer cells compared to all other groups at 24 and 48 h, even surpassing the equivalent dose of the free drug. This phenomenon could be attributed to the enhanced attachment and absorption of cancer cells to the high surface area of nanofibrous scaffolds that mimic the ECM structure, along with the controlled and sustained release of the drug from the scaffolds [84, 85]. Furthermore, nanofibrous scaffolds enable the local delivery of therapeutic components to the target tissue, thereby increasing drug bioavailability and minimizing adverse effects on normal tissues [79, 81, 82, 86, 87].

### Apoptosis assessment

**Fig. 10:**
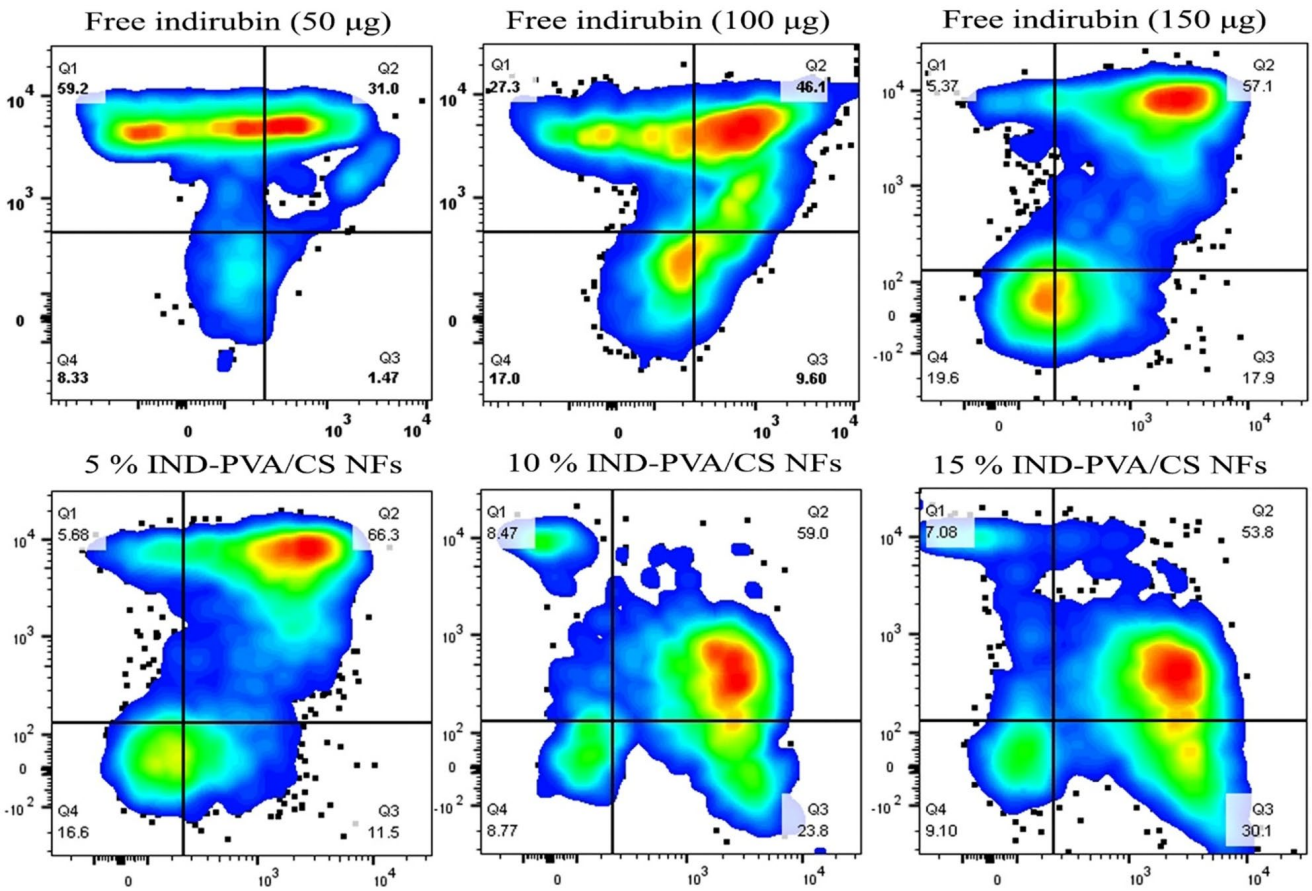
Analysis of apoptosis in A375 melanoma cells via flow cytometry following 24-hour treatment with various formulations

The results of apoptosis assessment indirubin-loaded nanofibrous scaffolds compared to an equivalent dose of the free drug of are represented as the scatter plots in Fig. 10. The apoptosis rates of treatments, the sum of early and late apoptosis (Q2 and Q3 quartelets in the scatter plots of Fig. 10) against A375 cells were about 32.47%, 55.7%, 75%, 77.8%, 82.8%, and 83.9%, for free indirubin drug at concentrations of 50 µg, 100 µg, 150 µg, 5%, 10%, and 15% indirubin-PVA/CS NFs, respectively. The apoptosis of A375 melanoma cells induced by indirubin-PVA/CS NFs (15 wt%) was nearly 84%, the highest among all groups after 24 h of incubation. The potential apoptotic activity of indirubin has been demonstrated in numerous studies to be enhanced by encapsulation into a nanofibrous scaffold, providing both protection and controlled release [88–91]. This enhancement resulted in increased induction of apoptosis. The controlled release and localized delivery of indirubin ensure high efficacy and minimal side effects, establishing these nanofibers as a valuable tool in cancer suppression [92–94]. The apoptosis assessment of indirubin-loaded PVA/CS nanofibrous scaffolds demonstrates their potential as an effective therapeutic platform for melanoma cancer treatment [2, 95]. Although the total amount of indirubin released from the scaffold after 24 h was lower than the equivalent free indirubin doses (50–150 µg per well), the indirubin-loaded PVA/CS scaffolds exhibited higher cytotoxicity. This effect arises from the localized and sustained drug release, ECM-like porous structure, and enhanced cell–scaffold interactions, which increase local indirubin concentration and uptake at the tumor cell interface. Additionally, encapsulation within the PVA/CS matrix protects indirubin from degradation and enhances its bioavailability, resulting in a more effective induction of apoptosis compared to free indirubin [96–98].

Moreover, live–dead staining could further visualize the distribution of viable and nonviable cells on the scaffold surface. The combination of MTT and Annexin-V/PI flow cytometry assays already provides consistent quantitative evidence of indirubin-induced cytotoxicity; therefore, live–dead staining will be included in future work to complement these results.

## Conclusion

This study successfully developed a topical scaffold composed of electrospun PVA/CS nanofibers loaded with the natural compound indirubin, aiming to improve melanoma cell inhibition through a non-invasive, targeted approach. The obtained NFs exhibited suitable nanometric dimensions and morphology, with efficient drug loading achieved via electrospinning without adverse interactions. The obtained electrospun NFs with 15 wt% indirubin demonstrated favorable physicochemical characteristics, sustained drug release, potent antioxidant activity, and significant cytotoxicity and apoptotic effects against melanoma cells in vitro. These promising in vitro results support the potential of indirubin-loaded PVA/CS nanofibrous scaffolds as a localized treatment strategy for cutaneous melanoma. However, further preclinical in vivo studies are essential to validate therapeutic efficacy, safety, and pharmacokinetics before progressing to clinical trials.

## Data Availability

All data included in this study are available upon request by contacting the corresponding author.
